# Impact and Influence of Urinary Incontinence on Physical Activity Levels

**DOI:** 10.1016/j.euros.2023.07.004

**Published:** 2023-08-25

**Authors:** Rocío Adriana Peinado-Molina, Sergio Martínez-Vázquez, Antonio Hernández-Martínez, Juan Miguel Martínez-Galiano

**Affiliations:** a Department of Nursing, University of Jaen, Jaen, Spain; b Department of Nursing, Physiotherapy and Occupational Therapy, Ciudad Real Faculty of Nursing, University of Castilla-La Mancha, Ciudad Real, Spain; c Consortium for Biomedical Research in the Epidemiology and Public Health Network (CIBERESP), Madrid, Spain

**Keywords:** Urinary incontinence, Exercise, Women's health services, Pelvic floor disorders, Pelvic floor

## Abstract

**Background:**

The benefits of physical activity are numerous on both physical and mental levels. Urinary incontinence (UI) can influence physical activity level; among US women, nearly two out of three view this problem as a barrier to physical activity, meaning that they do not exercise, exercise less, or even have to change their activity routines to accommodate this pelvic floor dysfunction.

**Objective:**

To determine whether UI influences the pattern of physical activity and whether a greater impact of urinary symptoms could influence the level of physical activity.

**Design, setting, and participants:**

An observational study was carried out with women in 2021 and 2022 in Spain.

**Outcome measurements and statistical analysis:**

The main dependent variable was level physical activity, as measured by the International Physical Activity Questionnaire (IPAQ). The Urogenital Distress Inventory (UDI-6) scale was used to determine the presence of UI and its impact. Sociodemographic, health status, lifestyle and obstetric data were obtained. Bivariate and multivariate analyses were performed using binary logistic regression, obtaining adjusted odds ratio (aOR) with its 95% confidence interval (95% CI).

**Results and limitations:**

A total of 1446 women participated, of whom 55.8% (807) had UI and 25.7% (371) reported low physical activity. Mixed incontinence (aOR: 1.53; 95% CI: 1.09–2.15) overall and a greater intensity of urinary symptoms (UDI-6 score; aOR: 1.014; 95% CI: 1.01–1.02) in the group of women with incontinence were statistically associated with a higher frequency of low physical activity. Other variables related to low physical activity were age, body mass index, pelvic pain, and income level (*p* < 0.001).

**Conclusions:**

Mixed-type UI is associated with low-level physical activity or inactivity in the whole group of women, while among women with UI, the greater impact of the symptoms increases the probability of low physical activity or inactivity.

**Patient summary:**

In this report, it is analyzed how urinary incontinence affects physical activity. It was found that women who suffer from mixed-type urinary incontinence have a low level of physical activity or inactivity, while those who experience a greater impact of urinary incontinence symptoms have an increased likelihood of having low physical activity or inactivity.

## Take Home Message

Women do not exercise, exercise less, or even have to change their activity routines to accommodate the urinary incontinence, one of pelvic floor dysfunctions.

Mixed-type urinary incontinence is associated with low-level physical activity or inactivity in the whole group of women.

The greater impact of the urinary incontinence symptoms increases the probability of low physical activity or inactivity.

## 1 Introduction

The World Health Organization (WHO) defines physical activity as bodily movement produced by skeletal muscles requiring energy expenditure, thus referring to any activity, including leisure time, itinerary transport to work, or even intense physical activity improving health [1], [2]. Physical activity is a protective factor against different pathologies and health problems, and people who do not participate in physical activity are up to 30% more likely to die [1].

Physical activity has numerous benefits on both physical and mental levels [2], [3], [4], [5], [6]. On a physical level, it improves bone mass and resistance [3], and helps prevent cardiovascular diseases such as hypertension, weight loss [4], cancer [7], and diabetes [8]. On a mental level, it reduces the symptoms of depression and anxiety [5], [6], and improves thinking, learning, critical judgment, quality of life, and general well-being [5], [6]. These benefits occur in women at all stages of their lives [9].

The new WHO guidelines recommend at least 150–300 min of moderate or vigorous aerobic physical activity per week for all adults, including people living with chronic conditions or disability [10]. The consequences of physical inactivity at the health level and the cost that this entails, globally it would be approximately $5.2 billion in physical inactivity over a period of 11 yr (2020–30) if global levels of physical activity do not increase [11]. In this sense, it is necessary to know the physical activity patterns of women [12], as well as the factors associated with them [13], [14]. Thus, factors such as age [15], sex [16], socioeconomic level [17], or interest in the activity to be carried out, especially in women, are associated with the pattern of physical activity [18].

Other more specific factors can also influence the level of physical activity, such as urinary incontinence (UI). Among US women, nearly two out of three view this problem as a barrier to physical activity, meaning that they do not exercise, exercise less, or even have to change their activity routines to accommodate this pelvic floor dysfunction [19], [20], [21]. This is due, among other reasons, to urine loss during physical activity, which determines not to carry it out [22], [23]. Additionally, physical activity is considered a social activity; therefore, being affected by UI would negatively impact women's relationships and social life [24].

The pelvic floor is made up of muscles, ligaments, and fascia. This composition gives it stability to fulfill, among others, its urinary continence function; disturbance of this anatomical structure can cause UI [25]. Other pelvic floor dysfunctions include pelvic organ prolapse, pelvic pain [26], or fecal incontinence [27], [28], [29]. It is a prevalent problem worldwide and is underdiagnosed [27], [28], with global figures that, in some cases, reach 46.5% [25], [30], [31]. There are different studies with contrasting results, and very few deal with physical activity and its association with UI [32].

Owing to the high prevalence of incontinence and the consequences that it could have on the pattern of physical activity, it becomes a problem with a significant impact on the health of women on physical, mental, and social levels. For this reason and due to the scarcity of recent studies, the objective was to determine whether UI influences the pattern of physical activity and whether a greater impact of urinary symptoms could influence level physical activity.

## 2 Patients and methods

### 2.1 Design and patient selection

This observational study was conducted with women in 2021 and 2022 in Spain. Women under 18 yr of age were excluded, as well as those who had difficulty understanding Spanish, those who had given birth within the previous 12 mo, and those with mental health or cognitive disorders that could affect data collection.

To estimate the sample size, we used a prevalence of 11.6% [20], [21] for women who do not partake in physical activity within the group of women with UI, a confidence level of 95%, an absolute error of 3%, and an increase due to losses/dropouts of 10%. Thus, a minimum of 487 women with UI would be needed. Taking into account that UI can reach 46.5% [25], [30], [31] a total of 1047 women would be needed.

### 2.2 Information sources and study variables

First, women were recruited for the study, and informed consent was obtained. Next, trained observers interviewed the women included in the study to obtain sociodemographic and employment data, previous medical history, health status, lifestyle and habits, obstetric history, and health problem using a previously piloted self-made questionnaire. Data were collected consecutively.

To assess the impact of urinary symptoms, the Urogenital Distress Inventory (UDI-6) subscale of the Pelvic Floor Distress Inventory (PFDI-20) was used [33], [34]. Each question uses the following 0–4 response format, categorized into four levels of dysfunction: none, a little, moderate, or a lot. The minimum score for each subscale is 0, and the maximum is 100 points. The UDI-6 measures the presence, severity, associated urogenital symptoms, and type of UI. Presence and severity are measured on a Likert scale: 0, the symptom does not appear; 1, there is a symptom without discomfort; 2, a symptom with little discomfort; 3, a symptom with moderate discomfort; and 4, a symptom with a lot of discomfort. The presence of incontinence corresponds to values 1–4. This scale is converted into another from 0 to 100; the average value is found (quotient between the sum of values obtained and the number of items answered) and multiplied by 25, obtaining a result of 0, indicating null severity, to 100, indicating maximum severity of urogenital symptoms. Some of the items from the questionnaire are used to classify the type of incontinence. For example, item 2 identifies urge UI, item 3 identifies stress incontinence, and both identify mixed incontinence.

The International Physical Activity Questionnaire (IPAQ) was used in its short version to study physical activity [35], [36]. This classifies adult populations based on activity levels (low, moderate, and high). This questionnaire has adequate validity [35]. A low level of physical or inactive activity was considered when 600 METs (physical activity unit of the test) were not reached.

### 2.3 Statistical analysis

First, descriptive statistics were carried out using absolute and relative frequencies, and means with standard deviation (SD) for continuous variables. Next, a bivariate analysis was performed to determine the differentiating characteristics in the sociodemographic and clinical profile of the participating women in relation to the presence of UI.

Bivariate and multivariate analyses were then performed to determine the relationship between UI types and low physical activity or inactivity levels. Finally, this same analysis was carried out, but only on the population of women with incontinence and introducing the impact of urinary symptoms as an independent variable (UDI-6 scale). In both analyses, the Pearson chi-square test was used, and the odds ratio (OR) and adjusted odds ratio (aOR) were estimated with their respective 95% CIs, using binary logistic regression in the latter. When the multivariate analysis was carried out, it was decided to include all the variables with statistical association and/or considered potential confounding factors to adjust the relationship between the types of UI and the impact (UDI-6) on low-level physical activity or inactivity. The statistical program used to analyze the information was SPSS 28.0.

### 2.4 Ethical considerations

The study was approved by the Research Ethics Committee of the province of Jaen (SPCV-0220/0302-N-20). Before starting the questionnaire, the women had to read an information sheet about the study and its objectives, and confirm their consent to participate.

## 3 Results

A total of 1446 women participated. Their mean age was 44.3 yr (SD = 14.68) and the mean body mass index (BMI) was 25.0 (SD = 4.75). The majority, 85.7% (1239) of women, did not smoke, and 54.4% (786) drank occasionally. Regarding personal and obstetric history, 28.9% (418) were in menopause, 33.0% (477) had some type of illness, and 78.6% (1131) had been pregnant. Regarding the type of birth, 67.2% (917) had a previous vaginal birth and 26.2% (379) an instrumental one. Regarding the existence of UI and its types, we found that 55.8% (807) of the women reported having some type of UI, the most frequent being mixed in 26.3% (380). The rest of the characteristics can be found in Table 1.

**Table 1:** Sociodemographic and clinical characteristics of the study sample

Variable	*n* (%) *N* = 1446	Mean (SD)
Age (yr)		44.3 (14.68)
<30	220 (15.2)	
30–49.9	799 (55.3)	
≥50	427 (29.5)	
BMI		25.0 (4.75)
Normal weight <25	830 (57.4)	
Overweight 25–29.9	405 (28.0)	
Obesity ≥30	211 (14.6)	
Income level (€)		
<1000	196 (13.6)	
1000–1999	512 (35.4)	
2000–2999	425 (29.4)	
>3000	313 (21.6)	
Alcohol consumption		
Never	351 (24.3)	
Occasionally	786 (54.4)	
Only weekends	144 (10.0)	
Frequently	142 (9.8)	
Daily	23 (1.6)	
Smoking habit		
No	1239 (85.7)	
Yes	207 (14.3)	
Pregnancy		
None	315 (21.8)	
One	194 (13.4)	
Two or more	937 (64.8)	
Vaginal birth		
None	475 (32.8)	
One	289 (20.0)	
Two or more	682 (47.2)	
Instrumental birth		
No	1067 (73.8)	
Yes	379 (26.2)	
Tear		
No	904 (62.5)	
Yes	542 (37.5)	
Macrosomia		
No	1250 (86.4)	
Yes	194 (13.4)	
Menopause		
No	1028 (71.1)	
Yes	418 (28.9)	
Illness		
No	969 (67.0)	
Yes	477 (33.0)	
Cardiovascular disorder		
No	1323 (91.5)	
Yes	123 (8.5)	
Respiratory disorder		
No	1408 (97.4)	
Yes	38 (2.6)	
Endocrine disorder		
No	1298 (89.8)	
Yes	148 (10.2)	
Gynecological disorder		
No	1404 (97.1)	
Yes	42 (2.9)	
Musculoskeletal disorder		
No	1353 (93.6)	
Yes	93 (6.4)	
Neurological disorder		
No	1412 (97.6)	
Yes	34 (2.4)	
Neoplastic disease		
No	1434 (99.2)	
Yes	12 (0.8)	
Gastrointestinal disorder		
No	1404 (97.1)	
Yes	42 (2.9)	
Dermatological disorder		
No	1424 (98.5)	
Yes	22 (1.5)	
Neoplastic disorder		
No	1421 (98.3)	
Yes	25 (1.7)	
Nephrourological disorder		
No	1436 (99.3)	
Yes	10 (0.7)	
Immunological disorder		
No	1435 (99.2)	
Yes	11 (0.8)	
Ophthalmological ENT disorder		
No	1421 (98.3)	
Yes	25 (1.7)	
Type of incontinence		
No incontinence	639 (44.2)	
Exclusively stress incontinence	86 (5.9)	
Exclusively urge incontinence	341 (23.6)	
Mixed incontinence	380 (26.3)	

BMI = body mass index; ENT = ear, nose, and throat; SD = standard deviation.

Next, the differentiating characteristics between the women who reported having UI and those who did not present this problem were studied. In this analysis, it was observed that women with UI had a statistically significant (*p* ≤ 0.05) older age, higher BMI, greater number of pregnancies, vaginal births, instrumental birth, perineal trauma, higher frequency of menopausal status, higher percentage of previous illness, and greater probability of low physical activity (Table 2).

**Table 2:** Characteristics of the women based on the presence of urinary incontinence (bivariate analysis)

Variable	Urinary incontinence		*p* value
	No *n* (%) (*N* = 639)	Yes *n* (%) (*N* = 807)	
Age (yr)			**<0.001**
<30	145 (22.7)	75 (15.2)	
30–49.9	348 (54.5)	451 (55.9)	
≥50	146 (22.8)	281 (34.8)	
BMI			**<0.001**
Normal weight <25	430 (67.3)	400 (49.6)	
Overweight 25–29.9	157 (24.6)	248 (30.7)	
Obesity ≥30	52 (8.1)	159 (19.7)	
Income level (€)			0.618
<1000	78 (12.2)	118 (14.6)	
1000–1999	229 (35.8)	283 (35.1)	
2000–2999	191 (29.9)	234 (29.0)	
>3000	141 (22.1)	172 (21.3)	
Alcohol consumption			0.372
Never	139 (21.8)	212 (26.3)	
Occasionally	355 (55.6)	431 (53.4)	
Only weekends	68 (10.6)	76 (9.4)	
Frequently	66 (10.3)	76 (9.4)	
Daily	11 (1.7)	12 (1.5)	
Smoking habit			0.255
No	540 (84.5)	699 (86.6)	
Yes	99 (15.5)	108 (13.4)	
Pregnancy			**<0.001**
None	208 (32.6)	107 (13.3)	
One	95 (14.9)	99 (12.3)	
Two or more	336 (52.6)	601 (74.5)	
Vaginal birth			**<0.001**
None	301 (47.1)	174 (21.6)	
One	104 (16.3)	185 (22.9)	
Two or more	234 (36.6)	448 (55.5)	
Previous cesarean			0.127
None	523 (81.8)	660 (81.8)	
One	76 (11.9)	113 (14.0)	
Two or more	40 (6.3)	34 (4.2)	
Instrumental birth			**<0.001**
No	517 (80.9)	550 (68.2)	
Yes	122 (19.1)	257 (31.8)	
Perineal trauma (episiotomy or tear)			**<0.001**
No	534 (86.5)	565 (72.5)	
Yes	83 (13.5)	214 (27.5)	
Menopause			**<0.001**
No	492 (47.9)	536 (52.1)	
Yes	147 (35.2)	271 (64.8)	
Illness			**<0.001**
No	969 (67.0)		
Yes	477 (33.0)		
Cardiovascular disorder			**<0.001**
No	610 (95.5)	713 (88.4)	
Yes	29 (4.5)	94 (11.6)	
Respiratory disorder			0.113
No	627 (98.1)	781 (96.8)	
Yes	12 (1.9)	26 (3.2)	
Endocrine disorder			**0.030**
No	586 (91.7)	712 (88.2)	
Yes	53 (8.3)	95 (11.8)	
Gynecological disorder			0.623
No	622 (97.3)	782 (96.9)	
Yes	17 (2.7)	25 (3.1)	
Musculoskeletal disorder			**<0.001**
No	616 (96.4)	737 (91.3)	
Yes	23 (3.6)	70 (8.7)	
Neurological disorder			**0.014**
No	631 (98.7)	781 (96.8)	
Yes	8 (1.3)	26 (3.2)	
Neoplastic disease			0.447
No	635 (94.2)	799 (99.0)	
Yes	4 (0.6)	8 (1.0)	
Gastrointestinal disorder			**0.003**
No	630 (98.6)	774 (95.9)	
Yes	9 (1.4)	33 (4.1)	
Dermatological disorder			0.239
No	632 (98.9)	792 (98.1)	
Yes	7 (1.1)	15 (1.9)	
Neoplastic disorder			**0.014**
No	634 (99.2)	787 (97.5)	
Yes	5 (0.8)	20 (2.5)	
Nephrourological disorder			0.122
No	637 (99.7)	799 (99.0)	
Yes	2 (0.3)	8 (1.0)	
Immunological disorder			0.600
No	635 (99.4)	800 (99.1)	
Yes	4 (0.6)	7 (0.9)	
Ophthalmological ENT disorder			**0.004**
No	635 (99.4)	786 (97.4)	
Yes	4 (0.6)	21 (2.6)	
Prolapse			**<0.001**
No	588 (92.0)	655 (81.2)	
Yes	51 (8.0)	152 (18.8)	
Pelvic pain			**<0.001**
No	563 (88.1)	612 (75.8)	
Yes	76 (11.9)	195 (24.2)	
Low levels of physical activity			**0.022**
No	494 (77.3)	581 (72.0)	
Yes	145 (639)	226 (28.0)	

BMI = body mass index; ENT = ear, nose, and throat.

The values in bold are statistically significant values.

The next step was to determine the relationship between UI and low level of physical activity using bivariate and multivariate analyses. In this last analysis, it was observed that only the highest level of economic income was associated with a lower probability of low physical activity (*p* < 0.001), while pelvic pain (aOR: 1.48; 95% CI: 1.08–2.02) and the type of mixed incontinence (aOR: 1.53; 95% CI: 1.09–2.15) were statistically associated with a higher frequency of low physical activity (Table 3).

**Table 3:** Type of incontinence and relationship with low levels of physical activity (bivariate and multivariate analyses)

Variable	Low levels of physical activity		Bivariate analysis		Multivariate analysis	
	No *n* (%) (*N* = 1075)	Yes *n* (%) (*N* = 371)	OR (95% CI)	*p* value	aOR (95% CI)	*p* value
Age (yr)				**0.038**		0.177
<30	163 (74.1)	57 (25.9)	1 (ref.)		1 (ref.)	
30–49.9	613 (76.7)	186 (23.3)	0.87 (0.62–1.22)	0.418	0.73 (0.43–1.23)	0.230
≥50	299 (79.0)	128 (30.0)	1.22 (0.85–1.77)	0.278	1.12 (0.53–2.36)	0.774
BMI				**<0.001**		0.480
Normal weight <25	645 (77.7)	185 (22.3)	1 (ref.)		1 (ref.)	
Overweight 25–29.9	291 (71.9)	114 (28.1)	**1.37 (1.04–1.79)**	**0.024**	0.88 (0.55–1.43)	0.623
Obesity ≥30	139 (65.9)	72 (34.1)	**1.81 (1.30–2.51)**	**<0.001**	1.37 (0.61–3.10)	0.448
Income level (€)				**<0.001**		**<0.001**
<1000	116 (59.2)	80 (40.8)	1 (ref.)		1 (ref.)	
1000–1999	370 (72.3)	142 (27.7)	**0.56 (0.39–0.79)**	**<0.001**	**0.64 (0.44–0.92)**	**0.017**
2000–2999	334 (78.6)	91 (21.4)	**0.40 (0.27–0.57)**	**<0.001**	**0.46 (0.31–0.69)**	**<0.001**
>3000	255 (81.5)	58 (18.5)	**0.33 (0.22–0.49)**	**<0.001**	**0.39 (0.25–0.61)**	**<0.001**
Alcohol consumption				0.164		0.684
Never	244 (69.5)	107 (30.5)	1 (ref.)		1 (ref.)	
Occasionally	594 (75.6)	192 (24.4)	**0.74 (0.56–0.98)**	**0.032**	0.86 (0.64–1.16)	0.317
Only weekends	112 (77.8)	32 (22.2)	0.65 (0.41–1.03)	0.064	0.79 (0.48–1.26)	0.306
Frequently	109 (76.8)	33 (23.2)	0.69 (0.44–1.08)	0.107	0.83 (0.52–1.34)	0.451
Daily	16 (69.6)	7 (30.4)	1.00 (0.40–2.50)	0.996	1.34 (0.52–3.48)	0.549
Smoker				0.878		0.619
No	922 (74.4)	317 (25.6)	1 (ref.)		1 (ref.)	
Yes	153 (73.9)	54 (26.1)	1.03 (0.73–1.44)		1.09 (0.77–1.55)	
Menopause				0.119		0.087
No	776 (75.5)	252 (24.5)	1 (ref.)		1 (ref.)	
Yes	299 (71.5)	119 (28.5)	1.23 (0.94–1.58)		0.61 (0.34–1.08)	
Number of pregnancies				0.323		0.403
Zero	244 (77.5)	71 (22.5)	1 (ref.)		1 (ref.)	
One	140 (72.2)	54 (27.8)	1.33 (0.88–2.00)	0.178	1.36 (0.68–2.73)	0.391
Two or more	691 (73.7)	246 (26.3)	1.22 (0.91–1.65)	0.190	1.01 (0.45–2.27)	0.972
Number of vaginal births				0.157		0.649
Zero	369 (77.7)	106 (22.3)	1 (ref.)		1 (ref.)	
One	210 (72.7)	79 (27.3)	1.31 (0.94–1.83)	0.117	1.32 (0.72–2.42)	0.366
Two or more	496 (72.7)	186 (27.3)	1.31 (0.99–1.72)	0.057	1.01 (0.45–2.27)	0.395
Previous cesarean				0.508		0.482
None	873 (73.8)	310 (26.2)	1 (ref.)		1 (ref.)	
One	147 (77.8)	42 (22.2)	0.81 (0.56–1.16)	0.245	0.89 (0.55–1.43)	0.623
Two or more	55 (74.3)	19 (25.7)	0.97 (0.57–1.67)	0.920	1.37 (0.61–3.10)	0.448
Previous instrumental birth				0.230		0.554
No	802 (75.2)	265 (24.8)	1 (ref.)		1 (ref.)	
Yes	273 (72.0)	106 (28.0)	1175 (0.90–1.53)		0.91 (0.67–1.24)	
Perineal trauma				**0.030**		0.964
No	863 (75.6)	278 (24.4)	1 (ref.)		1 (ref.)	
Yes	212 (69.5)	93 (30.5)	**1.36 (1.03–1.80)**		1.01 (0.73–1.40)	
Menopause				0.118		0.087
No	776 (75.5)	252 (24.5)	1 (ref.)		1 (ref.)	
Yes	299 (71.5)	119 (28.5)	1.22 (0.95–1.58)		0.61 (0.34–1.08)	
Pelvic pain				**<0.001**		**0.015**
No	901 (76.7)	274 (23.3)	1 (ref.)		1 (ref.)	
Yes	174 (64.2)	97 (35.8)	**1.83 (1.38–2.43)**		**1.48 (1.08–2.02)**	
Prolapse				**0.002**		0.135
No	942 (75.8)	301 (24.2)	1 (ref.)		1 (ref.)	
Yes	133 (65.5)	70 (34.5)	**1.65 (1.20–2.26)**		1.30 (0.92–1.85)	
Previous illness				**0.024**		0.870
No	738 (76.2)	231 (23.8)	1 (ref.)		1 (ref.)	
Yes	337 (79.6)	140 (29.4)	**1.33 (1.04–1.70)**		1.02 (0.77–1.35)	
Type of incontinence				**<0.001**		**0.038**
No incontinence	494 (77.3)	145 (22.7)	1 (ref.)		1 (ref.)	
Exclusively stress incontinence	70 (81.4)	16 (18.6)	0.78 (0.44–1.38)	0.505	0.75 (0.42–1.35)	0.335
Exclusively urge incontinence	253 (74.2)	88 (25.8)	1.19 (0.87–1.61)	0.141	1.22 (0.89–1.67)	0.223
Mixed incontinence	258 (67.9)	122 (32.1)	**1.61 (1.21–2.14)**	**<0.001**	**1.53 (1.09–2.15)**	**0.014**

aOR = adjusted odds ratio; BMI = body mass index; CI = confidence interval; OR = odds ratio; ref. = reference.

The values in bold are statistically significant values.

**Fig. 1:**
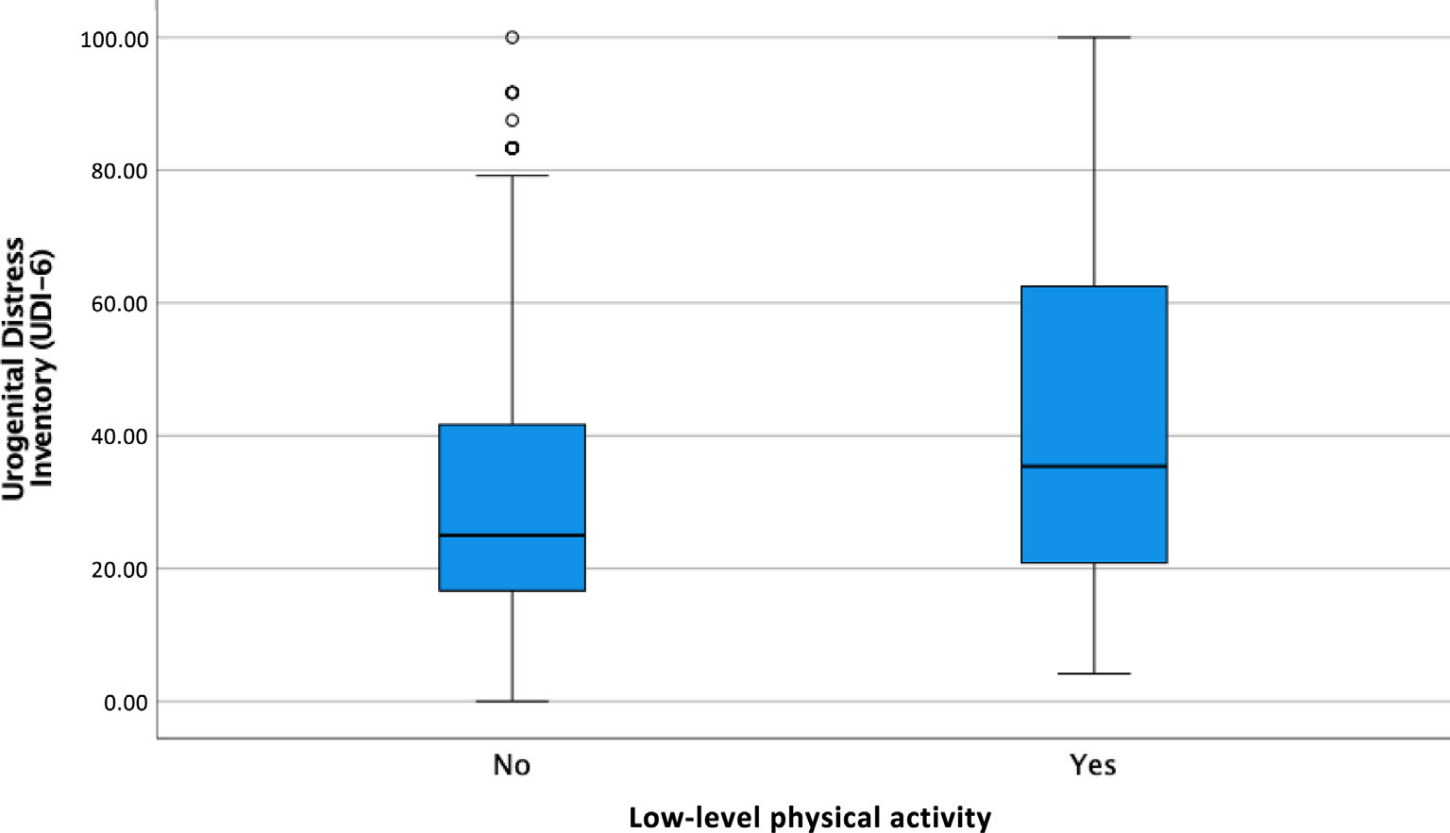
Urogenital Distress Inventory (UDI-6) scale scores according to low level of physical activity VS normal level physical activity.

Finally, it was analyzed how the impact of urinary symptoms influences low-level physical activity; for this analysis, only the group of incontinent women were included. In this way, after adjusting for all the variables, it was identified that a greater intensity of urinary symptoms (UDI-6 score) was associated with a greater probability of low physical activity (aOR: 1.014; 95% CI: 1.01–1.02; Table 4 and Fig. 1). Furthermore, in this analysis, older age and BMI were also associated with a higher probability of low physical activity, while a higher income level was associated with a lower probability of low physical activity (*p* < 0.001; Table 4).

**Table 4:** Factors associated with low levels of physical activity in women with urinary incontinence and its relationship with the impact of urinary symptoms (bivariate and multivariate analyses)

Variable	Low levels of physical activity		Bivariate analysis		Multivariate analysis	
	No *n* (%) (*N* = 581)	Yes *n* (%) (*N* = 226)	OR (95% CI)	*p* value	aOR (95% CI)	*p* value
Age (yr)				**0.001**		**0.045**
<30	58 (77.3)	17 (22.7)	1 (ref.)		1 (ref.)	
30–49.9	343 (76.1)	108 (23.9)	1.07 (0.60–1.92)	0.809	1.22 (0.55–2.71)	0.629
≥50	180 (64.1)	101 (35.9)	**1.91 (1.06–3.46)**	**0.032**	**2.99 (1.05–8.56)**	**0.041**
BMI				**0.001**		0.120
Normal weight <25	309 (77.3)	91 (22.8)	1 (ref.)		1 (ref.)	
Overweight 25–29.9	173 (69.8)	75 (30.2)	**1.47 (1.03–2.11)**	**0.034**	1.22 (0.83–1.79)	0.313
Obesity ≥30	99 (62.3)	60 (37.7)	**2.06 (1.38–3.06)**	**<0.001**	**1.59 (1.02–2.48)**	**0.040**
Income level (€)				**<0.001**		**0.003**
<1000	60 (50.8)	58 (49.2)	1 (ref.)		1 (ref.)	
1000–1999	205 (72.4)	78 (27.6)	**0.39 (0.25–0.61)**	**<0.001**	**0.51 (0.32–0.30)**	**0.007**
2000–2999	181 (77.4)	53 (22.6)	**0.30 (0.19–0.49)**	**<0.001**	**0.39 (0.23–0.24)**	**<0.001**
>3000	135 (78.5)	37 (21.5)	**0.28 (0.17–0.47)**	**<0.001**	**0.41 (0.23–0.73)**	**0.002**
Alcohol consumption				0.079		0.737
Never	137 (64.6)	75 (35.4)	1 (ref.)		1 (ref.)	
Occasionally	323 (74.9)	108 (25.1)	**0.61 (0.43–0.87)**	**0.007**	0.81 (0.55–1.19)	0.287
Only weekends	58 (76.3)	18 (23.7)	0.57 (0.31–1.03)	0.063	0.89 (0.46–1.69)	0.711
Frequently	55 (72.4)	21 (27.6)	0.70 (0.39–1.24)	0.220	0.93 (0.50–1.74)	0.824
Daily	8 (66.7)	4 (33.3)	0.91 (0.27–3.13)	0.885	1.57 (0.43–5.79)	0.500
Smoker				0.455		0.973
No	500 (71.5)	199 (28.5)	1 (ref.)		1 (ref.)	
Yes	81 (75.0)	27 (25.0)	0.84 (0.53–1.33)		0.99 (0.61–1.63)	
Menopause				0.054		0.054
No	401 (74.8)	135 (25.2)	1 (ref.)		1 (ref.)	
Yes	180 (66.4)	91 (33.6)	0.48 (0.23–1.01)		0.48 (0.23–1.01)	
Number of pregnancies				0.483		0.733
Zero	82 (76.6)	25 (23.4)	1 (ref.)		1 (ref.)	
One	69 (69.7)	30 (30.3)	1.43 (0.77–2.65)	0.262	1.05 (0.39–2.86)	0.915
Two or more	430 (71.5)	171 (28.5)	1.30 (0.81–2.11)	0.279	0.81 (0.28–2.37)	0.704
Number of vaginal births				0.159		0.605
Zero	135 (77.6)	39 (22.4)	1 (ref.)		1 (ref.)	
One	133 (71.9)	52 (28.1)	1.35 (0.84–2.19)	0.216	1.50 (0.68–3.33)	0.317
Two or more	313 (69.9)	135 (30.1)	1.49 (0.99–2.25)	0.055	1.46 (0.58–3.68)	0.427
Previous cesarean				0.097		0.218
None	466 (70.6)	194 (29.4)	1 (ref.)		1 (ref.)	
One	91 (80.5)	22 (19.5)	**0.58 (0.35–0.95)**	**0.031**	0.65 (0.35–1.19)	0.163
Two or more	24 (70.6)	10 (29.5)	1.00 (0.47–2.13)	0.998	1.33 (0.46–3.84)	0.604
Instrumental birth				0.398		0.226
No	401 (72.9)	149 (27.1)	1 (ref.)		1 (ref.)	
Yes	180 (70.0)	77 (30.0)	1.15 (0.83–1.60)		0.79 (0.53–1.16)	
Perineal trauma				0.127		0.739
No	432 (73.5)	156 (26.5)	1 (ref.)		1 (ref.)	
Yes	149 (68.0)	70 (32.0)	1.30 (0.93–1.83)		0.93 (0.63–1.40)	
Menopause				**0.012**		0.063
No	401 (74.8)	135 (25.2)	1 (ref.)		1 (ref.)	
Yes	180 (66.4)	91 (33.6)	**1.50 (1.09–2.07)**		0.49 (0.23–1.04)	
Pelvic pain				**<0.001**		0.205
No	464 (75.8)	148 (24.2)	1 (ref.)		1 (ref.)	
Yes	117 (60.0)	78 (40.0)	**2.09 (1.49–2.94)**		1.32 (0.86–2.04)	
Prolapse				**0.037**		0.764
No	482 (73.6)	173 (26.4)	1 (ref.)		1 (ref.)	
Yes	99 (65.1)	53 (34.9)	**1.49 (1.02–2.17)**		1.07 (0.70–1.64)	
Previous illness				**0.014**		0.750
No	371 (75.1)	123 (24.9)	1 (ref.)		1 (ref.)	
Yes	210 (67.1)	103 (32.9)	**1.48 (1.08–2.02)**		0.94 (0.65–1.37)	
Impact of urinary symptoms (UDI-6), mean (SD)	31.00 (20.42)	42.64 (27.38)	**1.02 (1.01–1.03)**	**0.001**	**1014 (1.01–1.02)**	**0.001**

aOR = adjusted odds ratio; BMI = body mass index; CI = confidence interval; OR = odds ratio; ref. = reference; SD = standard deviation; UDI-6 = Urogenital Distress Inventory.

The values in bold are statistically significant values.

## 4 Discussion

More than half of the women in the present study had UI, with mixed UI being the most frequent type and present in one out of four women. Among the factors associated with low physical activity in the group of women, low income, pelvic pain, and the type of UI were found. In addition, within the group of incontinent women, we found age, income, BMI, and the impact of urinary symptoms to have a relationship with a greater probability of low physical activity.

Among the strengths of the study, we can highlight the large sample size and the study of the impact of urinary symptoms, and not only the presence or absence of incontinence as a factor. In addition, to determine the physical activity level and presence of pelvic floor dysfunction, we used validated instruments that have already been used in a population similar to that of the study [33], [34], [35], [36]. In the case of a survey, memory and selection biases cannot be ruled out completely; however, if present in this study, there would be little influence on the results as the questionnaire had been piloted and the questions were formulated in colloquial language accessible to all educational levels. We do not believe that a confounding bias has influenced the results. We aimed to control for biases as much as possible with the selection criteria of the participants, for example, by excluding women who had given birth in the last 12 mo to avoid the influence that the process of pregnancy and childbirth can have on their pattern of physical activity, as well as by using a multivariable analysis and including in the model all those variables that could influence the results.

An older age was associated with a higher probability of low physical activity, in line with other authors [15], [37]. This may be due to physical deterioration with increasing age, which in turn is combined with a decrease in physical activity, affecting a woman's physical condition [37].

A low-income level was associated with a higher probability of having low physical activity, which coincides with the findings of other studies [17]. In this sense, Martins et al. [15] found that having few financial resources decreases physical activity rather than increasing it, in line with our results.

Pelvic pain, in our results, was identified to be associated with a greater possibility of having low physical activity, in line with other studies, although most collect results from the stage of pregnancy, childbirth, and postpartum [38]. Other authors indicate physical activity as an intervention that favors the reduction of the intensity of pain caused by endometriosis, although more research is needed [39].

In our study, the intensity of urinary symptoms was associated with the probability of low physical activity, such that a higher intensity increases the probability of low physical activity, as already identified by other researchers [19], [20], [21]. Although this seems to be due to the fact that urine losses force women to adapt their physical activity [22], [23], in some cases, it conditions them so significantly that they abandon physical activity entirely, becoming completely inactive [19], [40]. A priori, the bidirectionality of these variables, UI and physical activity, can be considered. However, not all women who have little physical activity have UI. We carried out a literature search and did not find any study that has studied these variables and considered this association: associating low physical activity with a greater probability of incontinence. In addition, UI has been identified in women who practice professional sports [41]. On the contrary, it is very reasonable to consider that women who have UI can stop doing physical activity. In the literature, it is stated that individuals who suffer from UI are more predisposed to suffer emotional problems and social isolation, and many feel ashamed and worried. Some even go so far as to drastically modify their habitual customs, especially in the case of women, avoiding leaving their homes, not using public transport, or rejecting sexual activity, among others [42]. Women with UI are afraid of urine leaking in social situations due to this lack of control and loss of self-esteem, and there is a social stigma regarding the subject. Women report feeling ashamed that urine leakage occurs, for example, when doing physical activity, and that it can be perceived by other people (the smell, the mark it can leave on clothes, etc.) [24]. All of this suggests that these reasons may lead women with UI to reduce and even stop doing physical activity, as has already been discussed and identified by other authors [32], [43].

With an adequate assessment and individualized interventions, the quality of life of these women could be improved, allowing them to exercise, thereby providing them with the benefits of physical activity. UI is associated with a lower level of physical activity, and these women are deprived of the benefits physical activity provides in preventing and dealing with multiple pathologies [2], [3], [4], [5], [6], [7], [8], [9].

In the approach to pelvic floor problems, and more specifically to UI, it is necessary to take into account the inclusion of the decrease in physical activity that this entails, in order to be able to implement the necessary measures to address it and encourage the initiation or maintenance of physical activity, so that these women can benefit from all that physical activity provides on physical, mental, and social levels. For this reason, it is also necessary to provide greater visibility to this problem experienced by many women, implementing controls and monitoring at all levels to detect these conditions and provide early care with adequate and effective treatment [44].

## 5 Conclusions

Mixed-type UI is associated with low-level physical activity or inactivity in women, while among women with UI, a greater impact of the symptoms increases the probability of low physical activity or inactivity.

  ***Author contributions:*** All authors had full access to all the data in the study and take responsability for the integrity of the data and the accuracy of the data analysis.

  *Study concept and design*: Juan Miguel Martínez-Galiano.

*Acquisition of data*: Juan Miguel Martínez-Galiano, Rocio Adriana Peinado-Molina, Sergio Martínez-Vázquez.

*Analysis and interpretation of data*: Juan Miguel Martínez-Galiano, Antonio Hernández-Martínez, Rocio Adriana Peinado-Molina.

*Drafting of the manuscript*: Juan Miguel Martínez-Galiano, Rocio Adriana Peinado-Molina, Sergio Martínez-Vázquez, Antonio Hernández-Martínez.

*Critical revision of the manuscript for important intellectual content*: Juan Miguel Martínez-Galiano, Rocio Adriana Peinado-Molina, Antonio Hernández-Martínez, Sergio Martínez-Vázquez.

*Statistical analysis*: Antonio Hernández-Martínez.

*Obtaining funding*: Juan Miguel Martínez-Galiano, Rocio Adriana Peinado-Molina.

*Administrative, technical, or material support*: Juan Miguel Martínez-Galiano, Antonio Hernánedez-Martínez, Sergio Martínez-Vázquez, Rocio Adriana Peinado-Molina.

*Supervision*: Juan Miguel Martínez-Galiano.

*Other*: None.

  ***Financial disclosures:*** All authors certifies that all conflicts of interest, including specific financial interests and relationships and affiliations relevant to the subject matter or materials discussed in the manuscript (eg, employment/affiliation, grants or funding, consultancies, honoraria, stock ownership or options, expert testimony, royalties, or patents filed, received, or pending), are the following: None.

  ***Funding/Support and role of the sponsor:*** This project was cofunded by the Operative Program FEDER 2014–2020, and the Ministry of Economics and Knowledge of the Government of Andalucía ( 1380358). The third author received a Grant from the Program University Teacher Training, financed by the Ministry of Universities Government of Spain (FPU20/01567).

  ***Ethics statement:*** The study received a favorable opinion from the Research Ethics Committee of the province of Jaen (SPCV-0220/0302-N-20). Before starting the questionnaire, the women had to read an information sheet about the study and its objectives, and confirm their consent to participate. The consent form notified all participants of our intent to publish.

  ***Data sharing:*** Data are available upon reasonable request.

  ***Acknowledgments:*** The authors thanks all the participants of this study.
